# Protective Effects of Fucoidan against Hydrogen Peroxide-Induced Oxidative Damage in Porcine Intestinal Epithelial Cells

**DOI:** 10.3390/ani9121108

**Published:** 2019-12-10

**Authors:** Yue Li, Weimin Zhao, Li Wang, Yueping Chen, Hao Zhang, Tian Wang, Xiaoyang Yang, Fei Xing, Junshu Yan, Xiaomin Fang

**Affiliations:** 1 Institute of Animal Science, Jiangsu Academy of Agricultural Sciences, Nanjing 210014, China; liyue9032@hotmail.com (Y.L.); zhao_weimin1983@aliyun.com (W.Z.); wanglids@163.com (L.W.); yangxy95v1@163.com (X.Y.); xingjam@163.com (F.X.); junshu_2000@163.com (J.Y.); 2 College of Animal Science & Technology, Nanjing Agricultural University, Nanjing 210095, China; chenyp0321@163.com (Y.C.); zhanghao89135@163.com (H.Z.); tianwangnjau@163.com (T.W.)

**Keywords:** fucoidan, hydrogen peroxide, oxidative stress, antioxidant capacity, porcine intestinal epithelial cells

## Abstract

**Simple Summary:**

High levels of production in intensive farming systems make domestic animals like piglets particularly susceptible to oxidative stress, which is detrimental to intestinal homeostasis and function. It is of paramount importance to identify effective and reliable nutrients to counteract oxidative damage to the porcine intestinal epithelium, especially with the recent phasing out of the use of antibiotics in China. This study indicates that fucoidan could ameliorate hydrogen peroxide-induced oxidative stress in porcine intestinal epithelial cells, primarily owing to the action of fucoidan to facilitate nuclear factor-erythroid 2-related factor-2 signals and cellular antioxidant responses. These findings may provide useful implications for practical swine production.

**Abstract:**

This study was conducted to evaluate the effectiveness of fucoidan in ameliorating hydrogen peroxide (H_2_O_2_)-induced oxidative stress to porcine intestinal epithelial cell line (IPEC-1). The cell viability test was initially performed to screen out appropriate concentrations of H_2_O_2_ and fucoidan. After that, cells were exposed to H_2_O_2_ in the presence or absence of pre-incubation with fucoidan. Hydrogen peroxide increased the apoptotic and necrotic rate, boosted reactive oxygen species (ROS) generation, and disturbed the transcriptional expression of genes associated with antioxidant defense and apoptosis in IPEC-1 cells. Pre-incubation with fucoidan inhibited the increases in necrosis and ROS accumulation induced by H_2_O_2_. Consistently, in the H_2_O_2_-treated IPEC-1 cells, fucoidan normalized the content of reduced glutathione as well as the mRNA abundance of NAD(P)H quinone dehydrogenase 1 and superoxide dismutase 1 while it prevented the overproduction of malondialdehyde. Moreover, H_2_O_2_ stimulated the translocation of nuclear factor-erythroid 2-related factor-2 to the nucleus of IPEC-1 cells, but this increase was further promoted by fucoidan pre-treatment. The results suggest that fucoidan is effective in protecting IPEC-1 cells against oxidative damage induced by H_2_O_2_, which may help in developing appropriate strategies for maintaining the intestinal health of young piglets.

## 1. Introduction

The intestinal mucosa is a complex and dynamic tissue that consists of a single layer of epithelial cells (intestinal epithelium) and the underlying lamina propria [1]. Its primary function is to digest and absorb essential nutrients and to act as a natural barrier defense between the gut lumen and the rest of the body [2]. Aside from being exposed to luminal nutrients, the complex intestinal epithelium is in fact continuously challenged by various diet-derived oxidants as well as by endogenous reactive species or oxidants generated during oxidative metabolism [3]. Oxidative stress is defined as an imbalance between the excessive production of reactive oxygen species (ROS) and their elimination by antioxidants [4]. The intestinal tract is actually a major site of antioxidant action, rendering the intestinal epithelium sensitive and vulnerable to oxidative stress [5,6]. The renewal and destruction of intestinal epithelium is a tightly controlled process in adult mammals, and its rapid self-renewal is crucial for regulating intestinal homeostasis, maintaining mucosal integrity, and repairing mucosal injury [7]. However, disruption of intestinal cellular homeostasis by redox signaling during oxidative stress can break the balance between renewal and death of intestinal epithelial cells and impair mucosal integrity, resulting in various intestinal diseases, such as inflammatory bowel diseases and enteric infections [8]. The prevalent oxidative stress occurring in swine production is a common mechanism by which various stress stimulators damage intestinal homeostasis and function, ultimately leading to compromised growth performance and inferior health outcomes of pigs, as well as economic losses of farmers [9,10]. An in vitro model of the porcine gastrointestinal tract has been established using porcine intestinal epithelial cell lines, and the available literature has shown that oxidative stress causes detrimental damage to porcine intestinal epithelial cells by disturbing cellular antioxidant homeostasis, accelerating enterocyte apoptosis, impairing cell permeability, and altering cellular signal transduction pathways, such as the classic nuclear factor-erythroid 2-related factor-2 (NRF2) signal pathway [11,12,13,14].

It is imperative that effective and reliable antioxidants are sought to counteract oxidative damage to porcine intestinal epithelial cells, especially with the recent phasing out of the use of in-feed antibiotics in China, which are able to maintain the gut health of susceptive animals, such as swine and poultry. Algal polysaccharides have received growing attention in recent years due to their diverse biological functions, including antioxidant, anti-inflammation, anti-infection, and cancer prevention and therapy activities [15]. Fucoidan is composed of a group of fucose-rich sulfated polysaccharides containing variable amounts of galactose, xylose, and glucuronic acid, and is widely available in brown seaweeds, such as *Sargassum* sp. and *Fucus* sp. [16]. Numerous studies have demonstrated that fucoidan isolated from different sources possesses excellent antioxidant activity in vitro by employing a series of assays, including α, α-diphenyl-β-picrylhydrazyl free radical scavenging assay, superoxide assay, and total antioxidant and reducing power assay [17,18]. In a cell culture study, Gao et al. [19] showed that fucoidan administration prevents hydrogen peroxide (H_2_O_2_)-induced apoptosis in PC12 cells (the rat adrenal pheochromocytoma line) by decreasing ROS accumulation. Likewise, Roy Chowdhury et al. [20] found that a bacterial fucose-rich polysaccharide protects human lung fibroblast cells against H_2_O_2_-induced apoptosis and necrosis by directly scavenging ROS. The biological function of fucoidan against oxidative stress has also been reported in mesenchymal stem cells [21], normal human hepatocytes [22], and mouse adipocytes [23]. Michel et al. [24] initially showed that fucoidan is totally excreted after oral administration, since it cannot be fermented by intestinal bacterial flora in humans. In contrast, later findings indicated that fucoidan can be absorbed in the intestinal tract by determining serum fucoidan concentrations, with its absorption rate in the intestine being around 0.6% [25,26]. These aforementioned findings, although inconsistent, together suggest that intestine is the major active of fucoidan. However, little is known about the biological effects of fucoidan in intestinal epithelial cells, irrespective of their sources and species. According to its biological function, we therefore hypothesized that fucoidan could exhibit protective effects in porcine intestinal epithelial cells subjected to oxidative stress, and then investigated the effectiveness of fucoidan administration in alleviating H_2_O_2_-induced oxidative damage to porcine intestinal epithelial cell line (IPEC-1).

## 2. Materials and Methods

### 2.1. Cell Culture

The IPEC-1 used in this study was derived from small intestinal epithelium isolated from a neonatal unsuckled piglet, and was kindly gifted by Dr. Jing Zhang (School of Animal Science and Nutritional Engineering, Wuhan Polytechnic University, Wuhan, Hubei, China). The IPEC-1 cells were cultured in Dulbecco’s modified eagle’s medium/nutrient mixture F-12 supplemented with 10% fetal bovine serum, 1% streptomycin-penicillin (100 U/mL), and 1% insulin-transferrin-selenium. The cell culture was grown and maintained at 37 °C in a 90% humidified atmosphere containing 5% carbon dioxide. The culture medium was changed daily and passaged every 2 days. All reagents used in the cell culture experiment were purchased from Gibco (Thermo Fisher Scientific Inc., Waltham, MA, USA).

### 2.2. Establishment of Oxidative Stress

Hydrogen peroxide (Sigma-Aldrich, St. Louis, MO, USA) was employed to induce oxidative stress to IPEC-1 cells in this study. The IPEC-1 cells were placed into a 96-well plate at a density of 1 × 10^4^ cells per well in 100 μL of culture medium and allowed to adhere overnight. The seeding medium was then removed and replaced with fresh medium containing varying concentrations of H_2_O_2_ (0, 0.1, 0.25, 0.5, 1.0, and 1.5 mM), which was incubated at 37 °C for 1 h according to a previous finding [27]. Each concentration was repeated six times in parallel. After that, 10 μL of Cell Counting Kit-8 (CCK-8) was added to each well to determine cell viability by measuring absorbance at 450 nm using a microplate reader (Thermo Fisher Scientific Inc., NY, USA) according to the instructions provided by the manufacturer (Dojindo, Tokyo, Japan). The result of the cell viability was expressed as the percentage of optical density of treated wells against vehicle-treated control wells, which were assigned a viability of 100%. The appropriate H_2_O_2_ concentration was then screened out for a later experiment according to the cell viability data.

### 2.3. Fucoidan Treatment

After selecting the concentration of H_2_O_2_, IPEC-1 cells (1 × 10^4^ cells/well in 96-well plate) were co-cultured with 0, 25, 50, 100, and 200 μg/mL fucoidan (*Undaria pinnatifida* fucoidan extract, purity ≥95%; Sigma-Aldrich, St. Louis, MO, USA) that was dissolved in phosphate-buffered saline (PBS) for 24 h before treatment with the above selected H_2_O_2_ solution for 1 h. The control wells were maintained with medium supplemented with an equivalent volume of PBS alone. Each treatment was performed in six replicate wells. The cell viability was determined as mentioned above, and the optimal administration level of fucoidan was then selected for subsequent use. According to the selected concentration of H_2_O_2_ and fucoidan, IPEC-1 cells were then treated with H_2_O_2_ solution for 1 h in the presence or absence of a 24-h pre-incubation with fucoidan. The control cells were treated with an equivalent volume of PBS vehicle alone. The cells were sampled for the determination of apoptosis and necrosis, intracellular ROS production, antioxidant-related parameters, gene expression, and nuclear NRF2 translocation.

### 2.4. Assay of Apoptosis and Necrosis

Cell apoptosis and necrosis were measured on a BD FACSCalibur flow cytometry system (BD Biosciences, San Jose, CA, USA) using the Alexa Fluor^®^ 488 Annexin V/Dead Cell Apoptosis Kit (Invitrogen Life Technologies, Gaithersburg, MD, USA) as described previously [28]. The IPEC-1 cells were placed into six-well plates. After the corresponding treatment, cells were harvested, washed twice with pre-cold PBS, and re-suspended in binding buffer containing Annexin V-fluorescein isothiocyanate and prodiumkono iodide, which was subsequently incubated in dark conditions for 15 min at 4 °C. The cell suspension was then ready for detection by flow cytometry assay.

### 2.5. Measurement of Intracellular ROS

The intracellular ROS level in IPEC-1 cells was determined using a ROS assay kit (Beyotime Institute of Biotechnology, Nantong, Jiangsu, China) according to the manufacturer’s instructions. Following each specific treatment, cells cultured in a 12-well plate were washed twice with PBS and incubated with the ROS dye (2′,7′-dichloro-dihydro-fluorescein diacetate) for 30 min. The ROS-positive cells labeled with green fluorescence were visualized with an Eclipse 80i inverted fluorescence microscope (Nikon Instruments, Tokyo, Japan), and fluorescence intensity was measured and quantified using Image J software (National Institute of Health, Bethesda, MD, USA) and normalized to the control value.

### 2.6. Determination of Antioxidant-Related Parameters

The IPEC-1 cells were cultured in six-well plates. After the corresponding treatment, cells were carefully scraped, re-suspended in 0.86% sterile saline, and disrupted by ultrasonication in an ice-cold water bath. The cell debris was removed by centrifugation and the clear supernatant was collected for subsequent determination. The activities of superoxide dismutase (SOD) and glutathione peroxidase (GPX) as well as the levels of the reduced form of glutathione (GSH) and malonaldehyde (MDA) in the supernatant were quantified using colorimetric kits in accordance with the manufacturer’s guidelines (Nanjing Jiancheng Bioengineering Institute, Nanjing, Jiangsu, China). Briefly, SOD activity was determined using the hydroxylamine method [29], and one unit of SOD was defined as the amount of enzyme that can produce a 50% inhibition of the rate of nitrite generation at 37 °C. The 5,5′-dithiobis (2-nitrobenzoic acid) (DTNB) method [30] was employed to determine GPX activity, one unit of which was calculated as the amount of enzyme that was required to deplete 1 μmol of GSH at 37 °C. The DTNB method [31] and thiobarbituric acid method [32] were used to determine the GSH and MDA concentrations, respectively. All acquired results were normalized against the total protein concentration in each sample for inter-sample comparison, and the total protein concentration was determined by the Bradford method [33], using crystalline bovine serum albumin (Sigma-Aldrich, St Louis, MO, USA) as the standard protein.

### 2.7. RNA Isolation and Reverse Transcription

Total RNA was isolated from fresh IPEC-1 cells using the TRIzol reagent kit (TaKaRa Biotechnology, Dalian, Liaoning, China) following the protocol provided by the manufacturer. The integrity of extracted RNA was verified by electrophoresis on 1.5% agarose gel, and its purity and concentration were quantified according to A_260/280_ readings using a NanoDrop ND-3000 UV-spectrophotometer (Nano Drop Technologies, Wilmington, DE, USA). The RNA samples were diluted with nuclease-free water to a concentration of 0.5 μg/μL. After that, 1 μg of total RNA was reverse transcribed into complementary DNA using a PrimeScriptTM RT reagent kit (TaKaRa Biotechnology, Dalian, Liaoning, China) in accordance with the manufacturer’s guidelines. The reverse transcription condition was set as 15 min at 37 °C and 5 s at 85 °C.

### 2.8. Quantitative Real-Time PCR Analysis 

The mRNA expression of target and reference genes *NRF2*, heme oxygenase 1 (*HO1*), NAD(P)H quinone dehydrogenase 1 (*NQO1*), superoxide dismutase 1 (*SOD1*), superoxide dismutase 2 (*SOD2*), glutathione peroxidase 1 (*GPX1*), glutathione S-transferase alpha 1 (*GSTA1*), catalase (*CAT*), proliferating cell nuclear antigen (*PCNA*), caspase 3 (*CASP3*), caspase 9 (*CASP9*), myeloid cell leukemia-1 (*MCL1*), B cell lymphoma 2 (*BCL2*), BCL2-associated X protein (*BAX*), glyceraldehyde-3-phosphate dehydrogenase (*GAPDH*), and beta actin (*ACTB*) were analyzed on an ABI StepOnePlus Real-Time PCR System (Applied Biosystems, Life Technologies, CA, USA). The primer sequences of the target and reference genes are presented in Table 1. The PCR mixture contained 2 μL of complementary DNA, 0.4 μL each of the forward and reverse primers, 10 μL of SYBR Premix Ex Taq (TaKaRa Biotechnology, Dalian, Liaoning, China), 0.4 μL of ROX reference dye (TaKaRa Biotechnology, Dalian, Liaoning, China), and 6.8 μL of nuclease-free water. The PCR procedures consisted of a pre-run at 95 °C for 30 s, 40 cycles of denaturation at 95 °C for 5 s, followed by a 60 °C annealing step for 30 s. The melting curve conditions were set up as follows: One cycle of denaturation at 95 °C for 10 s, followed by an increase in temperature from 65 to 95 °C with a temperature change rate of 0.5 °C/s. The 2^-ΔΔCT^ method [34] was employed to calculate the relative mRNA expression of the target genes (fold changes) after normalization against the reference gene, *ACTB*. The mRNA abundance of each target gene in the control cells was assigned an arbitrary value of 1.

### 2.9. Immunofluorescence Assay of NRF2

Nuclear NRF2 translocation was determined by the immunofluorescence assay as previously described by Zhang et al. [35], with minor modifications. Briefly, IPEC-1 cells were fixed in 4% paraformaldehyde solution in 0.1 M PBS at room temperature for 30 min after washing twice with PBS. The fixed cells were further permeabilized with 0.5% Triton X-100 for an additional 30 min. Following the washing with PBS, cells were incubated with the blocking buffer (5% bovine serum albumin in PBS) for 1 h at 4 °C before incubation with a rabbit anti-NRF2 antibody (1: 100, Proteintech Group, Inc., Chicago, IL, USA) in PBS at 4 °C overnight. After washing with PBS with 0.1% Tween 20, cells were incubated with a secondary antibody, Alexa Fluor 488-conjugated goat anti-rabbit immunoglobulin G (1: 100, Invitrogen, Carlsbad, CA, USA), in dark conditions for 1 h at 4 °C. The 5 µg/mL 4,6-diamidino-2-phenylindole was adopted for nuclear staining. Images were visualized with an Eclipse 80i inverted fluorescence microscope (Nikon Instruments, Tokyo, Japan), and nuclear NRF2 fluorescence intensity was calculated using the Image J software (National Institute of Health, Bethesda, MD, USA) and normalized against control cells.

### 2.10. Statistical Analysis

Data were analyzed by using SPSS 22.0 statistical software. Statistical differences between mean values were determined one-way analysis of variance (ANOVA) and Tukey’s post hoc test for multiple or pairwise comparison. A *p* value less than 0.05 was considered as statistically significant. Results are presented as mean ± standard error (SE) of at least three parallel measurements.

## 3. Results

### 3.1. Cytotoxic Effects of H_2_O_2_

**Figure 1:**
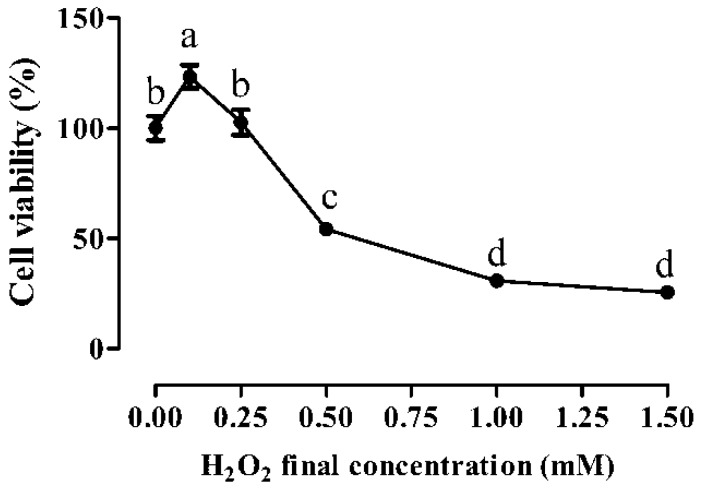
Cell viability of IPEC-1 cells as tested by CCK-8 dye after the treatments of different H_2_O_2_ concentrations. The data were calculated as a percentage of the control cells. Statistical differences between mean values were analyzed by ANOVA and Tukey’s post hoc test for multiple comparison. Values are means ± SE, n = 6. Different lowercase letters indicate statistically significant differences (*p* < 0.05).

Compared with the vehicle-treated control cells, low concentrations of H_2_O_2_ (0.1 and 0.25 mM) treatment for 1 h did not impair IPEC-1 cell growth (Figure 1), and actually the lowest concentration of H_2_O_2_ (0.1 mM) even stimulated cell growth (*p* < 0.05). In contrast, high levels of H_2_O_2_ exposure (from 0.5 to 1.5 mM) exhibited a dose-dependent inhibitory effect on IPEC-1 cell viability. In detail, cell viability was decreased by nearly 50% when exposed to 0.5 mM H_2_O_2_ for 1 h (*p* < 0.05), and dropped to around 25% when treated with 1.0 and 1.5 mM H_2_O_2_ for 1 h (*p* < 0.05). According to the median lethal dose, the 1-h exposure to 0.5 mM H_2_O_2_ was therefore selected as stress conditions in order to investigate the cytoprotective effect of fucoidan in IPEC-1 cells subjected to oxidative stress.

### 3.2. IPEC-1 Cell Viability in the Presence of Fucoidan

**Figure 2:**
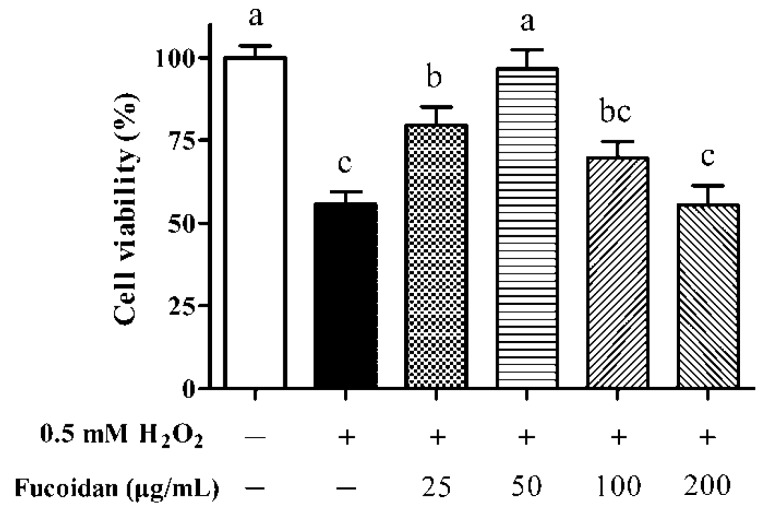
Effect of different fucoidan concentrations on the cell viability of IPEC-1 cells under the conditions of oxidative stress. The data were calculated as a percentage of the control cells. Statistical differences between mean values were analyzed by ANOVA and Tukey’s post hoc test for multiple comparison. Values are means ± SE, n = 6. Different lowercase letters indicate statistically significant differences (*p* < 0.05).

Compared with untreated cells, cell viability was decreased by approximately 50% after exposure to 0.5 mM H_2_O_2_ for 1 h (Figure 2, *p* < 0.05). The lowest concentration of fucoidan (25 μg/mL) administration increased the viability of IPEC-1 cells exposed to H_2_O_2_ (*p* < 0.05) but still did not reach the value observed in untreated cells (*p* < 0.05). The decrease in cell viability resulting from H_2_O_2_ treatment was totally reversed by 50 μg/mL fucoidan (*p* < 0.05), and its level was comparable with that in the control cells (*p* > 0.05). However, high concentrations of fucoidan treatment (100 and 200 μg/mL) did not exert protective effects on cell viability (*p* > 0.05). According to these results, 50 μg/mL fucoidan was therefore selected to investigate its effectiveness in relieving H_2_O_2_-induced oxidative damage to IPEC-1 cells.

### 3.3. Cell Apoptosis and Necrosis

**Figure 3:**
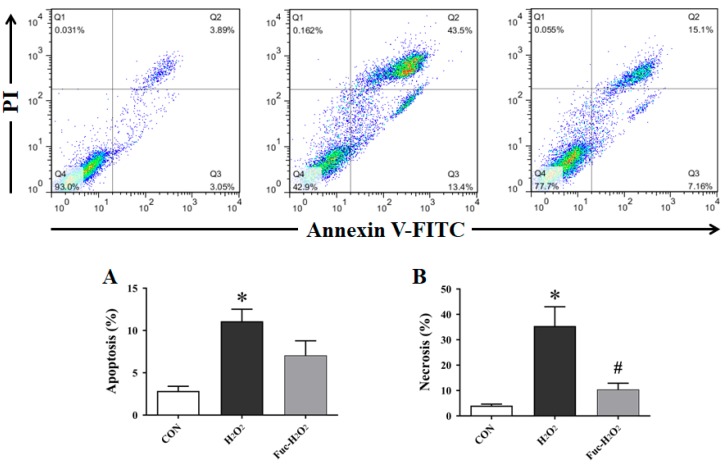
Effect of fucoidan on the rate of apoptosis (A) and necrosis (B) of IPEC-1 cells under the conditions of oxidative stress. In each plot, Q1 quadrant represents naked nucleus cells; Q2 quadrant represents necrotic cells; Q3 quadrant represents apoptotic cells; and Q4 quadrant represents viable cells. CON, cells were kept in culture medium; H_2_O_2_, cells were kept in culture medium and treated with 0.5 mM H_2_O_2_; Fuc-H_2_O_2_, cells were incubated in culture medium containing 50 μg/mL fucoidan and then treated with 0.5 mM H_2_O_2_. Statistical differences between mean values were analyzed by ANOVA and Tukey’s post hoc test for pairwise comparison. Values are means ± SE, n = 3. * Significant difference (*p* < 0.05) from the CON group. ^#^ Significant difference (*p* < 0.05) from the H_2_O_2_ group.

As shown in the Figure 3, the exposure to 0.5 mM H_2_O_2_ for 1 h markedly increased the apoptotic and necrotic rate in IPEC-1 cells when compared with the untreated cells (*p* < 0.05). On the contrary, the pre-incubation with 50 μg/mL fucoidan for 24 h significantly counteracted the increased necrosis induced by H_2_O_2_ stimulation (*p* < 0.05). However, a similar protective effect was not observed in IPEC-1 cell apoptosis (*p* > 0.05), even though it was numerically decreased by fucoidan administration.

### 3.4. ROS Level and Antioxidant Capacity

**Figure 4:**
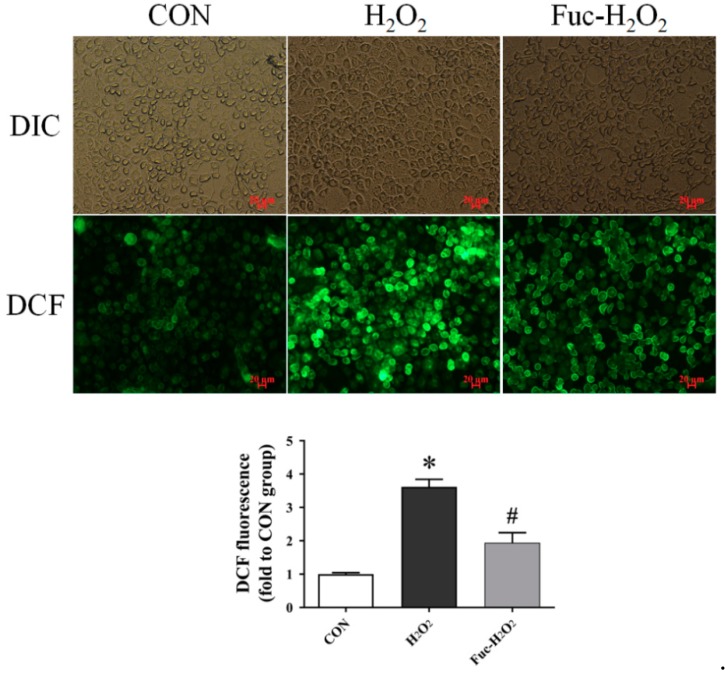
Effect of fucoidan on the ROS accumulation of IPEC-1 cells under the conditions of oxidative stress. The fluorescence intensity of 2′,7′-dichlorofluorescein (DCF) in IPEC-1 cells represented the intracellular accumulation of ROS measured using a fluorescence microscope. CON, cells were kept in culture medium; H_2_O_2_, cells were kept in culture medium and treated with 0.5 mM H_2_O_2_; Fuc-H_2_O_2_, cells were incubated in culture medium containing 50 μg/mL fucoidan and then treated with 0.5 mM H_2_O_2_. Statistical differences between mean values were analyzed by ANOVA and Tukey’s post hoc test for pairwise comparison. Values are means ± SE, n = 3. * Significant difference (*p* < 0.05) from the CON group. ^#^ Significant difference (*p* < 0.05) from the H_2_O_2_ group.

**Figure 5:**
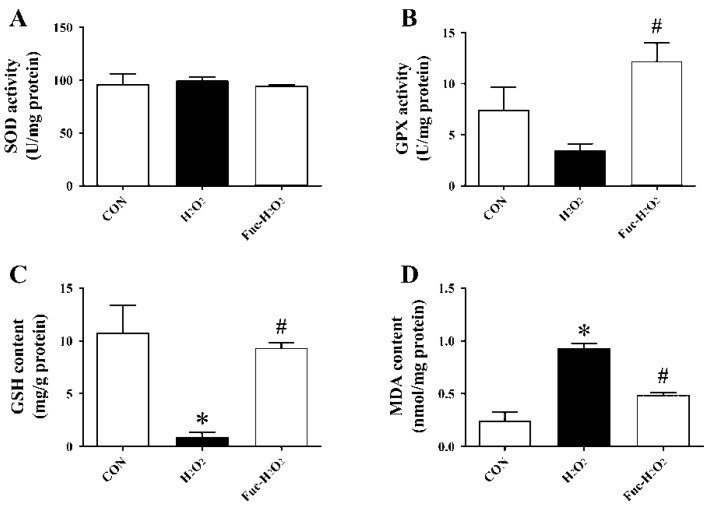
Effect of fucoidan on the activities of SOD (A) and GPX (B) and the contents of GSH (C) and MDA (D) of IPEC-1 cells under the conditions of oxidative stress. CON, cells were kept in culture medium; H_2_O_2_, cells were kept in culture medium and treated with 0.5 mM H_2_O_2_; Fuc-H_2_O_2_, cells were incubated in culture medium containing 50 μg/mL fucoidan and then treated with 0.5 mM H_2_O_2_. Statistical differences between mean values were analyzed by ANOVA and Tukey’s post hoc test for pairwise comparison. Values are means ± SE, n = 3. * Significant difference (*p* < 0.05) from the CON group. ^#^ Significant difference (*p* < 0.05) from the H_2_O_2_ group.

The treatment of 0.5 mM H_2_O_2_ for 1 h increased intracellular ROS production in the IPEC-1 cells as shown by an increase in fluorescence intensity (Figure 4, *p* < 0.05), and it was vastly decreased by pre-incubation with 50 μg/mL fucoidan for 24 h (*p* < 0.05). As indicated in Figure 5, exposure to H_2_O_2_ increased MDA accumulation but decreased GSH concentration in IPEC-1 cells (*p* < 0.05). In contrast, an elevated GSH concentration and decreased MDA content in IPEC-1cells subjected to H_2_O_2_ were observed when supplementing culture medium with 50 μg/mL fucoidan (*p* < 0.05). Moreover, the GPX activity of IPEC-1 cells cultured in medium containing fucoidan prior to H_2_O_2_ treatment was higher than those cells cultured with H_2_O_2_ alone (*p* < 0.05). The SOD activity was, however, similar among the three treatments (*p* > 0.05).

### 3.5. Gene Expression and the Nuclear Translocation of NRF2

**Figure 6:**
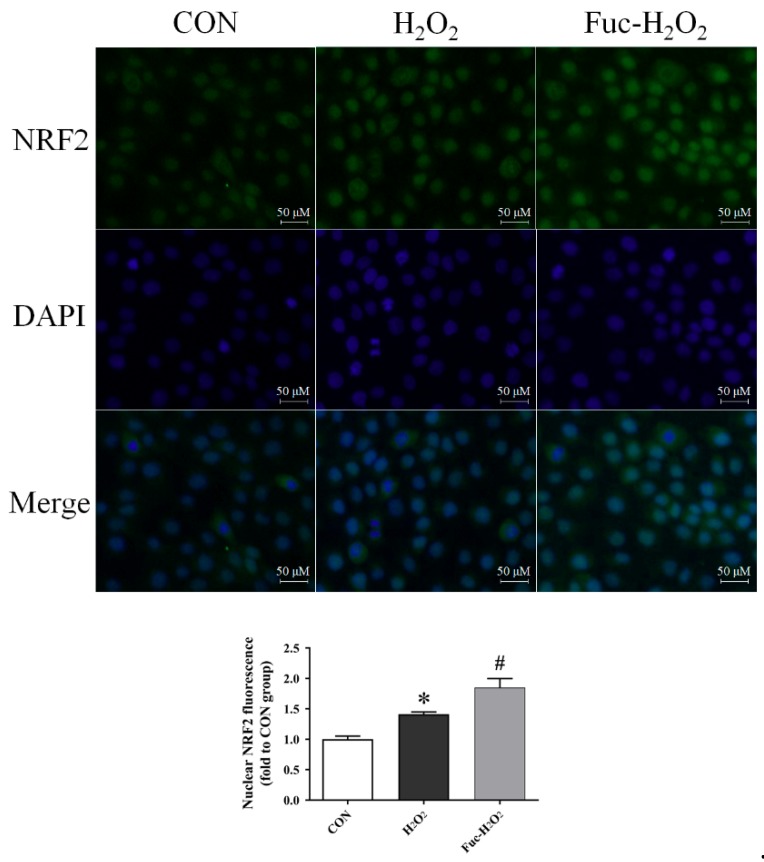
Effect of fucoidan on the level of nuclear NRF2 translocation of IPEC-1 cells under the conditions of oxidative stress. CON, cells were kept in culture medium; H_2_O_2_, cells were kept in culture medium and treated with 0.5 mM H_2_O_2_; Fuc-H_2_O_2_, cells were incubated in culture medium containing 50 μg/mL fucoidan and then treated with 0.5 mM H_2_O_2_. Statistical differences between mean values were analyzed by ANOVA and Tukey’s post hoc test for pairwise comparison. Values are means ± SE, n = 3. * Significant difference (*p* < 0.05) from the CON group. ^#^ Significant difference (*p* < 0.05) from the H_2_O_2_ group.

As indicated in Table 2, the exposure to 0.5 mM H_2_O_2_ for 1 h downregulated mRNA expression of *NQO1*, *SOD1*, and *MCL1* in IPEC-1 cells (*p* < 0.05). The H_2_O_2_ treatment also numerically increased *NRF2* mRNA expression level but did not reach a significant level (*p* = 0.056). The pre-treatment with fucoidan increased the mRNA abundance of *NQO1* and *SOD1* (*p* < 0.05), but a similar effect was not observed in *MCL1* mRNA expression (*p* > 0.05). Moreover, the mRNA level of *GPX1* was increased by pre-inoculation with fucoidan when compared with those cells subjected to H_2_O_2_ only (*p* < 0.05). Treatment did not affect the mRNA abundance of *HO1*, *SOD2*, *GSTA1*, *CAT*, *PCNA*, *CASP3*, *CASP9*, *BCL2*, or *BAX* (*p* > 0.05). Compared with the control cells, H_2_O_2_ stimulated the translocation of NRF2 to the nucleus of IPEC-1 cells (*p* < 0.05), according to the fluorescence intensity data presented in Figure 6. The increase in the translocation of NRF2 to the nucleus in the IPEC-1 cells exposed to H_2_O_2_ was further promoted by fucoidan administration (*p* < 0.05).

## 4. Discussion

Hydrogen peroxide, a source of ROS, can rapidly penetrate the cell membrane and generate highly toxic hydroxyl radicals by reacting with intracellular transition metals, and the exposure to H_2_O_2_ is a well-established method to induce oxidative stress in both cell cultures and animal tissues [36]. Cai et al. [12] showed that treatment with 0.5 to 1.0 mM H_2_O_2_ for 1 h is suitable to induce oxidative stress in IPEC-J2 cells, according to the results of intracellular free radical production and cell viability. Similarly, Vergauwen et al. [11] also reported that incubating IPEC-J2 cells with 0.5 mM H_2_O_2_ for 1 h induces intracellular oxidative stress. In this study, the exposure to 0.5 to 1.5 mM H_2_O_2_ for 1 h resulted in oxidative stress in IPEC-1 cells, as shown by a sharp decrease in cell viability. The viability of IPEC-1 cells exposed to 0.5 mM H_2_O_2_ was downgraded by nearly 50%; therefore, this concentration was selected to establish an in vitro oxidative stress model in the current research. The exposure to 0.5 mM H_2_O_2_ induced increases in intracellular ROS production as well as cell apoptosis and necrosis in our study, further indicating that an oxidative stress model was successfully established. It is necessary to mention, however, that the lowest concentration of H_2_O_2_ (0.1 mM) actually stimulated cell growth in this study. Results in a variety of cultured mammalian cells have, in fact, demonstrated that low levels of ROS, such as superoxide and H_2_O_2_, stimulate cell growth and growth response by activating and/or inactivating the transcription of specific genes involved in cell cycle progression [37,38]. 

The highly sulfated structure endows fucoidan with prominent hydroxyl radical and superoxide anion scavenging activities, enabling its potential use as a natural antioxidant in the food industry especially in terms of a secondary antioxidant [39,40,41]. The pre-incubation with 25 and 50 μg/mL fucoidan for 24 h prevented H_2_O_2_-induced inferior cell viability in our study, and the protective effect was more pronounced when its concentration was 50 μg/mL. In agreement with our finding, a reduced viability in rat adrenal pheochromocytoma cells subjected to H_2_O_2_ was attenuated, when treating cells with 30 and 60 μg/mL fucoidan [19]. In the current research, however, the treatment of IPEC-1 cells that suffered oxidative stress with 100 or 200 μg/mL fucoidan actually did not exert a protective effect, which may be pertinent to the cytotoxicity of fucoidan at higher doses [23]. Likewise, Jeong et al. [42] observed that the administration of 50 μg/mL fucoidan alleviates 5-fluorouracil-induced cell growth inhibition in dendritic cells, but a high level of fucoidan (100 μg/mL) exhibits a slight cytotoxicity.

The intracellular ROS level is a sensitive biomarker to reflect the oxidative stress status. The increase in intracellular ROS production observed currently after exposure to H_2_O_2_ is associated with the toxic hydroxyl radicals generated from H_2_O_2_ [36]. Similar results have also been reported by Cai et al. [43] and Kahlert et al. [44] in porcine intestinal epithelial cells. The overproduction of ROS also impaired the antioxidant system in IPEC-1 cells, as supported by the simultaneously increased MDA accumulation (an end product of lipid peroxidation) and decreased GSH concentration (an important antioxidative substance). In this study, the pre-inoculation with 50 μg/mL fucoidan effectively reduced ROS production in IPEC-1 cells, and it preserved cellular antioxidant status by decreasing MDA accumulation and increasing GSH levels. The ability of fucoidan to protect the antioxidant system of IPEC-1 cells subjected to oxidative stress is correlated with its sulfated chemical structure that could directly scavenge ROS as well as its regulation of redox signals, such as the NRF2 signaling pathway, as summarized by Phull and Kim [18]. Of note, Wang et al. [39] also reported that fucoidan improves antioxidant capacity and reduces the levels of MDA and ROS in human hepatocytes under oxidative stress. 

Usually, uncontrolled and/or prolonged oxidative stress is detrimental to lipids, protein, and DNA, and results in cell cycle interruption and arrest, which can ultimately cause apoptosis and necrosis in intestinal epithelial cells via various signaling pathways [6]. In this study, we once again confirmed that oxidative stress induced by H_2_O_2_ caused serious apoptosis and necrosis in porcine intestinal epithelial cells, accompanied by a simultaneous decrease in the mRNA expression of *MCL1*, an important anti-apoptotic protein, which may be directly associated with the increased ROS generation resulting from the exposure to H_2_O_2_ [45]. In an in vitro study, Gao et al. [19] showed that fucoidan treatment inhibits apoptosis in H_2_O_2_-induced PC12 cells by increasing the BCL2/BAX ratio, decreasing active CASP3 expression, and facilitating protein kinase B phosphorylation. A similar study conducted on human lung fibroblast cells also revealed that fucoidan inhibits H_2_O_2_-induced cell apoptosis and necrosis, and the underlying mechanism includes inhibition of caspase activity, a decrease in the BAX/BCL2 ratio, relocalization of BAX and cytochrome c, upregulation of anti-apoptotic members of the BCL2 family, and a decline in the phosphorylation of mitogen-activated protein kinases [20]. In this study, fucoidan protected the IPEC-1 cells against H_2_O_2_-induced necrosis, but the expression levels of key genes involved in the apoptotic pathway (e.g., *CASP3*, *CASP9*, *BCL2*, and *BAX*) were almost similar among treatments. Despite this, we cannot exclude the participation of these apoptosis-associated regulators in the protective action of fucoidan to prevent the excessive necrosis of IPEC-1 cells induced by H_2_O_2_, since gene expression patterns do not always reflect their enzymatic or signal activities. Thus, additional work is required to determine the potential mechanisms by which fucoidan preserves cellular homeostasis under stressful conditions.

Nuclear factor erythroid 2-related factor 2 is a key nuclear transcription factor, which is ubiquitously expressed in a wide range of cell types, and it regulates a series of defensive genes that encode detoxifying enzymes and antioxidant proteins [46]. Inside the nucleus, NRF2 heterodimerizes with a cofactor molecule, musculo-aponeurotic fibrosarcoma, and then binds the antioxidant response element sequences to activate various target genes, such as *NQO1*, *HO1*, *SOD*, *GPX*, and *CAT* [47]. Under normal physical conditions, the inhibitor of NRF2 retains NRF2 in the cytoplasm and cytoplasmic NRF2 is continuously degraded, which in turn results in a relatively low accumulation of nuclear NRF2; however, NRF2 will translocate to nuclei to activate defensive genes under the conditions of oxidative stress [48]. The increased nuclear NRF2 fluorescence intensity in IPEC-1 cells subjected to H_2_O_2_ in the current study was therefore a cellular defense response. Recently, Zhuang et al. [49] also reported that H_2_O_2_ induces a translocation of NRF2 to the nuclei in rat small intestine epithelial cells. Contradictory with the increase in the nuclear NRF2 accumulation, the mRNA expression of *NQO1* and *SOD1* was downregulated in IPEC-1 cells during oxidative stress, and this may be due to cell antioxidant system failure. A similar result has also been reported recently, in which H_2_O_2_ upregulates cellular NRF2 protein levels but downregulates the mRNA expression of *SOD1* in IPEC-1 cells [50]. Studies have demonstrated that fucoidan prevents oxidative damage by activating the NRF2 signaling pathway in human hepatocytes [22] and human lung fibroblast cells [20]. In the present study, fucoidan also promoted the translocation of NRF2 to nuclei in IPEC-1 cells exposed to oxidative stress, as supported by its significantly high nuclear fluorescence intensity. The increase in nuclear NRF2 accumulation resulting from fucoidan is consistent with the reduced intracellular ROS production and improved antioxidant status of porcine intestinal epithelial cells subjected to oxidative stress induced by H_2_O_2_. Moreover, the mRNA levels of *NQO1*, *SOD1*, and *GPX1*, the downstream target genes of NRF2, were upregulated when treating cells with fucoidan under oxidative stress. These results together suggest that activation of NRF2 signals may be an underlying mechanism responsible for the antioxidant biological function of fucoidan in porcine intestinal epithelial cells facing oxidative stress.

## 5. Conclusions

In conclusion, our findings demonstrate that fucoidan could ameliorate H_2_O_2_-induced oxidative stress in porcine intestinal epithelial cells, and the mechanisms responsible for its protective effects may be associated with the activation of the NRF2 signaling pathway.

## Figures and Tables

**Table 1:** Primer sequences used for real-time PCR assay.

Name	Genbank ^1^	Sequence (5′→3′) ^2^	Length
*NRF2*	NM_001185152.1	GGACAGCAGAAGTGATCCCC	97
		CAAAACCGTATCACTGGCCG	
*HO1*	NM_001004027.1	TGATGGCGTCCTTGTACCAC	71
		GACCGGGTTCTCCTTGTTGT	
*NQO1*	NM_001159613.1	CATGGCGGTCAGAAAAGCAC	135
		ATGGCATACAGGTCCGACAC	
*SOD1*	NM_001190422.1	AAGGCCGTGTGTGTGCTGAA	118
		GATCACCTTCAGCCAGTCCTTT	
*SOD2*	NM_214127.2	GGCCTACGTGAACAACCTGA	126
		TGATTGATGTGGCCTCCACC	
*GPX1*	NM_214201.1	CCTCAAGTACGTCCGACCAG	85
		GTGAGCATTTGCGCCATTCA	
*GSTA1*	NM_214389.2	ACACCCAGGACCAATCTTCTG	199
		AGTCTCAGGTACATTCCGGG	
*CAT*	NM_214301.2	TCCAGCCAGTGACCAGATGA	182
		CCCGGTCAAAGTGAGCCATT	
*PCNA*	NM_001291925.1	TAGCCGCGTCGTTGTGATTC	105
		GGCCTCGTTGATGAGGTCTT	
*CASP3*	NM_214131.1	GGATTGAGACGGACAGTGGG	124
		CCGTCCTTTGAATTTCGCCA	
*CASP9*	XM_003127618.4	CTGCCAAGCAAATGGTCCAG	151
		ACAGGACATCCATCTGTGCC	
*MCL1*	NM_001348806.1	CGGAGTAACAAACTGGGGCA	177
		AACCCATCCCAGCCTCTTTG	
*BCL2*	XM_021099593.1	GAGTTCGGTGGGGTCATGTG	152
		TACAGCTCCACAAAGGCATCC	
*BAX*	XM_003127290.5	GGCCCTTTTGCTTCAGGGTTT	119
		GACACTCGCTCAACTTCTTGG	
*GAPDH*	NM_001206359.1	CCAAGGAGTAAGAGCCCCTG	125
		AAGTCAGGAGATGCTCGGTG	
*ACTB*	XM_003124280.5	TGGAACGGTGAAGGTGACAG	176
		CTTTTGGGAAGGCAGGGACT	

*NRF2*, nuclear factor, erythroid 2-related factor 2; *HO1*, heme oxygenase 1; *NQO1*, NAD(P)H quinone dehydrogenase 1; *SOD1*, superoxide dismutase 1; *SOD2*, superoxide dismutase 2; *GPX1*, glutathione peroxidase 1; *GSTA1*, glutathione S-transferase alpha 1; *CAT*, catalase; *PCNA*, proliferating cell nuclear antigen; *CASP3*, caspase 3; *CASP9*, caspase 9; *MCL1*, myeloid cell leukemia-1; *BCL2*, B cell lymphoma 2; *BAX*, BCL2-associated X protein; *GAPDH*, glyceraldehyde-3-phosphate dehydrogenase; *ACTB*, beta actin. ^1^ GenBank Accession Number. ^2^ Shown as the forward primer followed by the reverse primer.

**Table 2:** Effects of fucoidan pretreatment on the relative gene expression levels of IPEC-1 cells under the conditions of oxidative stress.

Items ^1^	CON	H_2_O_2_	Fuc-H_2_O_2_	Contrast	
				CON vs. H_2_O_2_	H_2_O_2_ vs. Fuc-H_2_O_2_
*NRF2*	1.00 ± 0.14	8.10 ± 1.99	8.97 ± 2.14	0.056	0.931
*HO1*	1.00 ± 0.04	1.01 ± 0.09	1.08 ± 0.12	0.997	0.821
*NQO1*	1.00 ± 0.07	0.22 ± 0.02 *	0.52 ± 0.07 ^#^	<0.001	0.024
*SOD1*	1.00 ± 0.08	0.58 ± 0.04 *	0.91 ± 0.08 ^#^	0.014	0.037
*SOD2*	1.00 ± 0.08	0.63 ± 0.07	1.06 ± 0.22	0.236	0.163
*GPX1*	1.00 ± 0.02	1.11 ± 0.10	1.86 ± 0.18 ^#^	0.797	0.011
*GSTA1*	1.00 ± 0.25	1.82 ± 0.42	1.75 ± 0.26	0.247	0.986
*CAT*	1.00 ± 0.03	0.93 ± 0.06	0.97 ± 0.08	0.729	0.892
*PCNA*	1.00 ± 0.06	0.97 ± 0.13	1.10 ± 0.18	0.988	0.784
*CASP3*	1.00 ± 0.28	1.04 ± 0.41	0.97 ± 0.37	0.997	0.991
*CASP9*	1.00 ± 0.08	1.04 ± 0.05	0.89 ± 0.07	0.920	0.341
*MCL1*	1.00 ± 0.09	0.60 ± 0.08 *	0.69 ± 0.04	0.020	0.668
*BCL2*	1.00 ± 0.12	0.68 ± 0.12	0.77 ± 0.15	0.280	0.867
*BAX*	1.00 ± 0.16	0.99 ± 0.17	1.09 ± 0.23	0.999	0.919

*NRF2*, nuclear factor erythroid 2-related factor 2; *NQO1*, NAD(P)H quinone dehydrogenase 1; *HO1*, heme oxygenase 1; *SOD1*, superoxide dismutase 1; *SOD2*, superoxide dismutase 2; *GPX1*, glutathione peroxidase; *GSTA1*, glutathione S-transferase alpha 1; *CAT*, catalase; *PCNA*, proliferating cell nuclear antigen; *CASP3*, caspase 3; *CASP9*, caspase 9; *MCL1*, myeloid cell leukemia-1; *BCL2*, B cell lymphoma 2; *BAX*, BCL2-associated X protein. ^1^ CON, cells were kept in culture medium; H_2_O_2_, cells were kept in culture medium and treated with 0.5 mM H_2_O_2_; Fuc-H_2_O_2_, cells were incubated in culture medium containing 50 μg/mL fucoidan and then treated with 0.5 mM H_2_O_2_. Values are means ± SE, n = 3. * Significant difference (*p* < 0.05) from the CON group. ^#^ Significant difference (*p* < 0.05) from the H_2_O_2_ group.
